# Mining Two Decades of Soybean Genomics Literature Using Rule-Based Text Mining: Chromosome-Resolved Patterns of *Glyma* Gene Mentions

**DOI:** 10.3390/ijms27083398

**Published:** 2026-04-10

**Authors:** My Abdelmajid Kassem, Dounya Knizia, Khalid Meksem

**Affiliations:** 1 Plant Genomics and Bioinformatics Lab, Department of Biological and Forensic Sciences, Fayetteville State University, Fayetteville, NC 28301, USA; 2 School of Agricultural Sciences, Southern Illinois University, Carbondale, IL 62901, USA; dounya.knizia@siu.edu (D.K.); meksem@siu.edu (K.M.)

**Keywords:** soybean, *Glycine max*, text mining, rule-based extraction, PubMed, gene mentions, chromosome-level analysis

## Abstract

Soybean (*Glycine max* [L.] Merr.) is a globally important crop with a rapidly expanding body of genomics literature driven by advances in sequencing and functional genomics. Thousands of studies reference soybean genes using standardized *Glyma* identifiers; however, systematic analyses of how these identifiers are distributed across chromosomes in the scientific literature remain limited. Here, we present a chromosome-resolved bibliometric analysis of soybean gene mentions using a reproducible rule-based text mining approach. PubMed abstracts published between December 2006 and December 2025 were mined for standardized *Glyma* gene identifiers using regular-expression-based entity extraction. A total of 377 PubMed records were retrieved, of which 340 abstracts (90.2%) contained at least one *Glyma* gene identifier. The median number of unique genes mentioned per abstract was 1, with a maximum of 14 genes reported in a single study. Our results reveal three major patterns. First, soybean genomics research remains predominantly gene-centric, with most abstracts referencing one or two genes. Second, apparent chromosome-level disparities exist in literature representation within the subset of studies using standardized *Glyma* identifiers, with chromosomes 3 and 16 exhibiting the highest frequencies of unique gene mentions. A Chi-square goodness-of-fit test confirmed that these differences deviate significantly from a uniform distribution (χ^2^ = 123.71, *p* < 0.001), indicating non-random patterns of gene reporting. Third, a small subset of genes dominates the literature, while the majority of annotated genes are mentioned infrequently, reflecting a long-tailed distribution of research attention. This analysis captures reporting patterns in studies that explicitly use standardized *Glyma* identifiers and therefore represents a defined subset of the broader soybean genomics literature. Within this scope, the findings highlight uneven adoption of standardized gene nomenclature and chromosome-level differences in research emphasis. More broadly, this study demonstrates the utility of transparent, rule-based text mining approaches for large-scale bibliometric analyses in plant science and provides a scalable framework for comparative analyses across crop species.

## 1. Introduction

Soybean (*Glycine max* [L.] Merr.) is one of the most economically and nutritionally important legume crops worldwide, serving as a primary source of plant-derived protein and oil for human consumption, animal feed, and numerous industrial applications [1]. Global soybean production has increased dramatically over the past several decades, driven by rising demand for protein-rich feedstocks and vegetable oils [2]. Beyond its agricultural significance, soybean also represents a model system for legume genomics due to its paleopolyploid genome, extensive genetic resources, and well-developed research community [3,4].

Improving soybean productivity and resilience has long been a major research priority. Breeding and molecular genetics efforts have focused on traits including yield stability, drought tolerance, salinity tolerance, disease resistance, flowering time regulation, nodulation efficiency, and seed composition [5,6,7]. The integration of quantitative trait locus (QTL) mapping, marker-assisted selection, and genomic selection has further accelerated trait improvement [8,9,10]. In parallel, functional genomics approaches—including transcriptomics, proteomics, and genome editing—have expanded our understanding of soybean gene regulation and biological pathways [11,12].

A major turning point in soybean research occurred with the publication of the soybean reference genome in 2010 [4]. This milestone enabled standardized gene annotation and the widespread adoption of the *Glyma* gene identifier system, in which each gene is assigned a chromosome number and a unique numeric identifier corresponding to its genomic position. The availability of chromosome-resolved assemblies and curated databases such as SoyBase has provided researchers with a unified framework for referencing soybean genes [13]. Subsequent updates to the soybean genome assembly and annotation have further refined gene models and improved genomic accuracy [14,15].

The standardization of gene nomenclature has had important implications for the scientific literature. Thousands of studies now reference *Glyma* gene identifiers when describing gene function, regulatory mechanisms, stress responses, and agronomic traits. The result is a rapidly expanding corpus of text containing structured gene identifiers embedded within unstructured narrative descriptions. While individual studies provide detailed biological insights, the literature as a whole represents an underexplored data source for understanding broader research trends.

Despite the abundance of soybean genomics publications, our knowledge of how genes are represented across the literature remains fragmented. Most research articles focus on individual genes, gene families, or specific biological pathways, leaving open the question of whether certain chromosomes or genomic regions receive disproportionate attention. Chromosome-level disparities in gene reporting could reflect biological factors such as gene density, duplication history, or clustering of trait-associated loci [4,16]. Alternatively, they may arise from sociotechnical influences, including historical research priorities, funding patterns, or the prominence of early-discovered genes.

Understanding such patterns is important for several reasons. First, uneven representation of genes across chromosomes may indicate potential blind spots in functional characterization. Second, bibliometric biases can shape the trajectory of future research by reinforcing attention on already well-studied genes [17]. Third, literature-level analyses may help identify underexplored genomic regions that could be relevant for crop improvement. However, systematic approaches for quantifying gene-level representation in plant genomics literature remain scarce.

Text mining provides a powerful framework for extracting structured information from large collections of unstructured text, including scientific abstracts and full-text articles [18,19,20,21]. In the biomedical domain, a wide range of computational approaches—ranging from rule-based systems to advanced machine learning methods—have been used for named entity recognition, relation extraction, and automated curation of gene and disease information [21,22,23]. Tools such as PubTator and related annotation platforms have demonstrated the feasibility of large-scale automated extraction of gene mentions from millions of articles [23].

While recent advances in machine learning and deep learning have improved performance in complex entity recognition tasks [24,25], rule-based approaches remain highly effective when standardized naming conventions are available [26]. The structured format of *Glyma* gene identifiers provides an ideal case for precise extraction using regular expressions, enabling reproducible and transparent gene-level text mining without the need for extensive model training or domain-specific tuning.

In plant science, computational text mining has primarily been applied to gene ontology curation, phenotype extraction, and knowledge base construction [27]. Comparatively fewer studies have investigated large-scale patterns of gene representation within the literature itself. Bibliometric analyses in other biological systems have shown that a small subset of genes often dominates research attention, while the majority remain understudied [17]. Whether similar patterns exist at the chromosome level in crop species such as soybean has not been systematically evaluated.

Furthermore, temporal dynamics in gene reporting may reflect technological and conceptual shifts in genomics research. The increasing accessibility of next-generation sequencing, transcriptome profiling, and genome editing technologies has likely influenced which genes are studied and reported over time [28,29]. A longitudinal analysis of gene mentions may therefore provide insight into the evolving landscape of soybean functional genomics.

To address these gaps, we conducted a chromosome-resolved analysis of soybean gene mentions in PubMed abstracts published between 2006 and 2025. Using a reproducible rule-based text mining approach implemented in Python (v3.10), we extracted standardized *Glyma* gene identifiers and quantified their distribution across chromosomes and publication years. By focusing on abstract-level reporting, we capture the primary reporting emphasis of each study within abstracts that explicitly use standardized *Glyma* identifiers while maintaining scalability across thousands of records.

Our objectives were threefold: (i) to quantify how soybean genes are distributed across chromosomes in the scientific literature; (ii) to evaluate temporal trends in chromosome-level gene reporting; and (iii) to identify the most frequently mentioned genes and assess the extent of literature concentration. Through this approach, we aim to provide a literature-scale perspective on soybean genomics research and demonstrate the value of transparent, rule-based text mining methods for large-scale analyses in plant science.

## 2. Results

### 2.1. Corpus-Level Distribution of Soybean Gene Mentions

**Figure 1:**
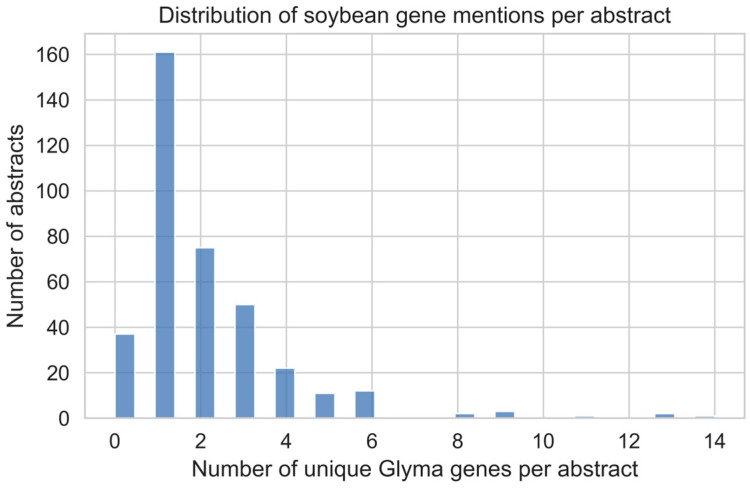
Distribution of unique soybean (*Glyma*) gene identifiers per PubMed abstract. The majority of abstracts mention one or two genes, with progressively fewer studies referencing larger gene sets, indicating a predominantly gene-centric research landscape.

The PubMed search yielded 377 records published between 2006 and 2025. Among these, 340 abstracts (90.2%) contained at least one *Glyma* gene identifier and were included in subsequent analyses. The distribution of unique *Glyma* gene identifiers per abstract was strongly right-skewed (Figure 1). The median number of unique genes mentioned per abstract was 1, with a maximum of 14 genes reported in a single study. The majority of abstracts referenced one or two soybean genes, while progressively fewer studies discussed larger gene sets. Only a small subset of publications contained more than five unique *Glyma* identifiers, indicating that most soybean genomics research remains gene-centric rather than genome-wide in scope.

This distribution reflects common experimental practices in soybean molecular biology, where studies often focus on individual candidate genes or small gene families associated with specific agronomic or physiological traits, rather than conducting comprehensive functional surveys across large genomic regions. The predominance of single-gene reporting further supports the suitability of abstract-level text mining as a means of capturing the principal biological emphasis of individual studies.

### 2.2. Chromosome-Level Distribution of Gene Mentions

**Figure 2:**
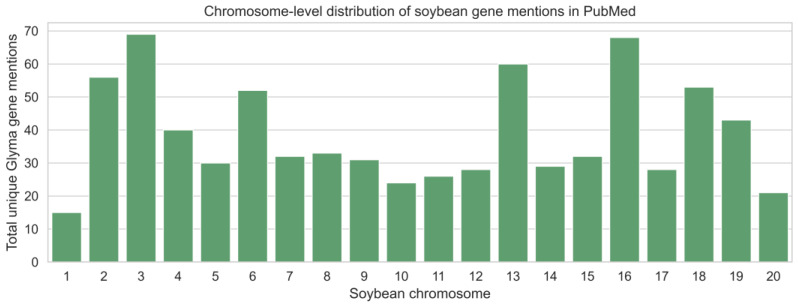
Chromosome-level distribution of unique soybean gene mentions aggregated across PubMed abstracts published between 2006 and 2025. Bars represent the total number of distinct *Glyma* gene identifiers associated with each chromosome.

Aggregating unique gene mentions across the entire corpus revealed substantial variation among soybean chromosomes (Figure 2). Chromosomes 2, 3, 6, 13, 16, and 18 exhibited the highest numbers of unique *Glyma* gene mentions (>50), whereas chromosomes 1, 10, 11, and 20 were consistently underrepresented (<25). These differences were not uniform across chromosomes. A Chi-square goodness-of-fit test confirmed that the distribution of gene mentions across chromosomes deviates significantly from a uniform distribution (χ^2^ = 123.71, *p* < 0.001), supporting the presence of non-random patterns of gene reporting within this corpus.

Notably, chromosomes with elevated gene mention counts are known to harbor loci associated with agronomically important traits, such as disease resistance, flowering time, and seed composition, as documented in SoyBase annotations. However, because this analysis reflects literature frequency rather than genomic content, these results likely represent a combination of biological relevance and historical research emphasis rather than underlying gene density alone.

### 2.3. Temporal Dynamics of Chromosome-Specific Gene Reporting

**Figure 3:**
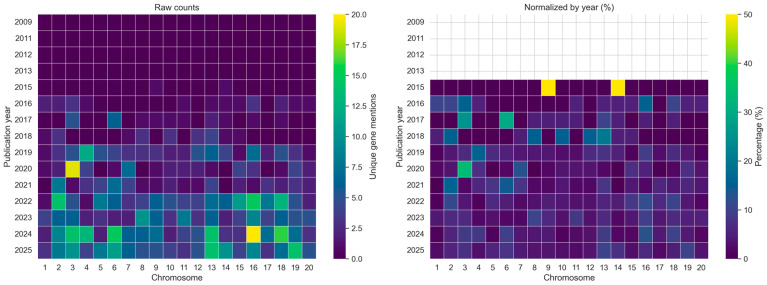
Temporal dynamics of soybean gene mentions across chromosomes. Heatmaps show (**left**) raw counts of unique *Glyma* gene identifiers mentioned per chromosome per year and (**right**) percentages normalized by the total number of gene mentions in each year, highlighting shifts in research focus over time.

To examine how soybean gene reporting has evolved over time, we analyzed chromosome-level gene mentions by publication year using both raw counts and normalized values (Figure 3). Prior to approximately 2015, soybean gene mentions were sparse and unevenly distributed across chromosomes. A marked increase in reporting was observed beginning around 2018, with a pronounced expansion particularly after 2020, where both absolute and relative increases are evident. This trend coincides with the maturation of soybean genomic resources, increased adoption of high-throughput sequencing technologies, and growing interest in climate-resilient crop traits.

To account for variation in publication volume across years, gene mention counts were additionally normalized as percentages of total mentions per year (Figure 3, right panel). The normalized heatmap confirms that the observed temporal patterns are not solely driven by increases in the number of publications but also reflect shifts in relative research emphasis across chromosomes.

Temporal heatmaps revealed that the increase in gene mentions was not uniform across chromosomes. Certain chromosomes, including chromosomes 3, 6, 13, 16, and 18, showed consistent enrichment in both absolute counts and normalized values in recent years, while others remained comparatively underrepresented. These findings indicate shifting research priorities and suggest that specific genomic regions have become focal points of soybean research in the past decade.

### 2.4. Most Mentioned Soybean Genes

**Figure 4:**
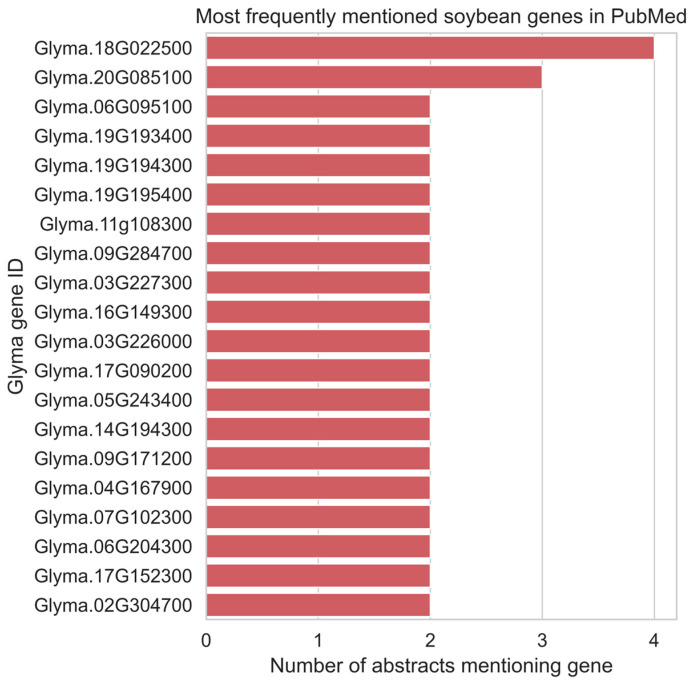
Most frequently mentioned soybean genes in PubMed abstracts. Bars represent the number of abstracts in which each gene is mentioned. Gene functional descriptions were obtained from SoyBase (www.soybase.org [13]).

At the gene level, analysis identified a small subset of *Glyma* genes that were repeatedly referenced across multiple abstracts (Figure 4). The most frequently mentioned genes were associated with well-characterized biological processes and agronomically relevant traits, including stress responses, disease resistance, and developmental regulation, as described in SoyBase gene annotations (Table 1). Many additional genes were mentioned with similarly low frequencies (e.g., two mentions).

In contrast, the majority of soybean genes appeared infrequently, often being mentioned in only one or two abstracts. This long-tailed distribution highlights a strong literature bias toward a limited number of well-studied genes and underscores the existence of a large pool of comparatively understudied soybean genes that may represent opportunities for future functional characterization.

## 3. Discussion

In this study, we present a chromosome-resolved, literature-scale analysis of soybean gene mentions using a reproducible rule-based text mining approach applied to PubMed abstracts. By extracting standardized *Glyma* gene identifiers from two decades of publications, we provide a quantitative overview of how soybean genes are represented across chromosomes and over time. To our knowledge, this work represents one of the first chromosome-resolved overviews of soybean gene reporting patterns across the scientific literature.

The corpus-level distribution of gene mentions indicates that soybean genomics research remains predominantly focused on individual genes or small gene sets. This gene-centric pattern is consistent with experimental traditions in plant molecular biology, where functional validation typically proceeds through candidate gene approaches, reverse genetics, and trait-specific investigations [6,11,12]. Although genome-wide technologies such as RNA-seq, GWAS, and whole-genome resequencing have become increasingly common [8,14,15], published abstracts often emphasize a limited number of key genes emerging from broader analyses. This tendency toward candidate-focused reporting is a well-documented feature of biological research communication [17].

Chromosome-level analysis revealed pronounced disparities in gene mention frequencies that cannot be readily explained by chromosome number alone. Soybean chromosomes differ in gene density, duplication history, and QTL distribution as a consequence of ancient polyploidization and segmental duplication events [3,4]. Chromosomes exhibiting higher literature representation—such as chromosomes 3 and 16—may harbor genes associated with widely studied agronomic traits, including disease resistance [30,31], stress responses [32,33], flowering regulation [34,35,36], and metabolic processes [13]. However, literature frequency does not necessarily correlate with intrinsic genomic importance. As shown in other organisms, research attention is often influenced by historical momentum, funding priorities, and early landmark discoveries [17,37]. Thus, the observed chromosome-level disparities likely reflect an interplay between biological relevance and sociotechnical research dynamics.

Temporal analyses further demonstrate that soybean genomics research has expanded markedly over the past decade, particularly following improvements in genome assemblies and pan-genome analyses [14,15]. The surge in chromosome-specific gene reporting after 2018 aligns with broader trends in plant genomics driven by next-generation sequencing, high-throughput transcriptomics, and CRISPR-based functional studies [28,29]. As technological barriers have decreased, researchers have gained access to increasingly precise genomic information, enabling more targeted gene discovery and characterization. The uneven growth of gene mentions across chromosomes suggests that emerging research priorities have concentrated attention on specific genomic regions, potentially leaving other regions comparatively underexplored.

At the gene level, the dominance of a small number of repeatedly studied genes reflects a common and well-characterized phenomenon in biomedical research. Large-scale bibliometric analyses have shown that a minority of genes accounts for a disproportionate share of scientific attention, while the majority remain understudied [17,38]. In soybean, frequently mentioned genes identified in this study are associated with core biological processes such as transcriptional regulation, oxidative stress response, and metabolic pathways, as documented in SoyBase annotations [13]. Meanwhile, the long-tailed distribution observed in our results suggests that many annotated soybean genes remain sparsely characterized in the literature, highlighting potential opportunities for functional genomics research.

Importantly, our findings illustrate the value of applying transparent, rule-based text mining approaches in plant science. Computational text mining has become an essential tool in biomedical informatics for extracting gene–disease associations, protein interactions, and curated knowledge from large text repositories [18,22,23]. While machine learning-based named entity recognition models such as BioBERT and deep neural architectures have demonstrated strong performance in complex biomedical contexts [24,25], rule-based approaches remain highly effective when standardized nomenclature is available [26]. The structured nature of *Glyma* identifiers enabled precise extraction with minimal ambiguity, illustrating that lightweight, interpretable methods can generate robust insights when applied to well-defined entity systems.

Beyond soybean, chromosome-level bibliometric mapping may offer a complementary lens for evaluating research distribution across plant genomes. As pan-genome analyses and comparative genomics expand across major crops [15,37,39], literature-scale mining approaches could help identify genomic regions that are underrepresented relative to their biological potential. Such analyses may inform funding strategies, prioritize candidate loci for functional validation, and support more balanced exploration of crop genomes.

The absence of widely studied genes commonly referred to by symbolic names (e.g., E1 or GmFT2a) further underscores the dependence of this analysis on standardized *Glyma* identifiers and highlights variability in gene naming conventions across the literature. Therefore, the observed patterns should be interpreted as reflecting reporting practices associated with standardized *Glyma* nomenclature rather than the full landscape of soybean genomics research.

Taken together, our results underscore that the scientific literature itself constitutes a structured dataset reflecting both biological knowledge and research behavior. By integrating genomic resources with transparent, rule-based text mining approaches, it becomes possible to quantify patterns of research emphasis at the genome scale [40,41]. While our approach does not infer biological causality, it provides a valuable meta-analytic perspective on how soybean genes are distributed within the scientific literature. As plant genomics continues to generate increasingly large and complex datasets, literature-scale analyses may serve as an important complement to experimental and computational genomics, offering insight into the evolving architecture of scientific attention.

Importantly, these findings reflect reporting patterns within a subset of the literature defined by standardized *Glyma* identifier usage and should not be interpreted as a comprehensive representation of soybean genomics research.

## 4. Materials and Methods

### 4.1. PubMed Corpus Collection

PubMed abstracts were retrieved using the National Center for Biotechnology Information (NCBI) E-utilities interface. Queries were constructed to identify records containing the term “*Glyma*” in the title or abstract and published between December 2006 and December 2025. This time window was selected to capture the period surrounding the release of the soybean reference genome and subsequent expansion of soybean genomics research. The PubMed query used was: (“*Glyma*” [Title/Abstract]) AND (“1 December 2006” [Date-Publication]: “31 December 2025” [Date-Publication]).

Search results were obtained using the ESearch utility with history tracking enabled, allowing batch retrieval of records via EFetch. Abstracts were downloaded in XML format and parsed using Python libraries to extract PubMed identifiers (PMIDs), publication years, article titles, and abstract text. Records lacking abstracts were retained for metadata completeness but excluded from text-based analyses. The PubMed query returned 377 records, of which 340 abstracts (90.2%) contained at least one standardized *Glyma* gene identifier and were included in downstream analyses.

### 4.2. Gene Name Recognition via Rule-Based Text Mining

Soybean gene mentions were identified using a rule-based text mining approach based on regular-expression matching. Specifically, we searched for standardized *Glyma* gene identifiers following the format “Glyma.xxGNNNNN”, where “xx” denotes the two-digit chromosome number (01–20) and “NNNNN” represents the gene number. Optional version suffixes (e.g., “.1”) were also captured.

This approach was chosen due to the highly standardized nature of *Glyma* gene nomenclature, which minimizes ambiguity and reduces the risk of false positives. Gene identifiers were extracted from the concatenated title and abstract text of each record. For each abstract, both the total number of gene mentions, and the number of unique gene identifiers were recorded.

### 4.3. Data Aggregation and Normalization

Extracted gene identifiers were mapped to their corresponding chromosomes based on the chromosome number embedded in the identifier. For each abstract, we computed chromosome-level counts of unique gene mentions. These counts were then aggregated across the entire corpus to generate chromosome-level summaries.

To examine temporal trends, publication years were parsed from PubMed metadata and used to group gene mentions by year. All analyses were performed in Python (v3.10) [42] using Jupyter notebooks (v8.6 [43], with data stored in CSV and pickle formats to ensure reproducibility. Data processing and aggregation were performed using the Pandas library (v2.2) [44], while visualization of results—including histograms, bar charts, and heatmaps—was carried out using Matplotlib (v3.10) [45] and seaborn (v0.13) [46]. The use of open-source tools ensured reproducibility and transparency of the analytical workflow.

## 5. Conclusions

This study presents, to our knowledge, one of the first chromosome-resolved, large-scale rule-based text mining analyses of soybean gene mentions in the biomedical literature. By systematically mining two decades of PubMed abstracts for standardized *Glyma* gene identifiers, we provide a quantitative overview of how soybean genes are reported across chromosomes within studies using standardized *Glyma* identifiers reported within studies using standardized *Glyma* identifiers across chromosomes and how reporting patterns have evolved over time.

Our results reveal three key findings: (i) soybean research remains largely gene-centric at the abstract level, with most studies focusing on one or a small number of genes; (ii) substantial chromosome-level disparities exist in gene reporting, indicating non-random research emphasis across the soybean genome; and (iii) a small subset of genes dominates the literature, while the majority of annotated genes remain infrequently mentioned. Together, these patterns highlight both the concentration and the uneven distribution of research attention within soybean genomics.

Beyond soybean specifically, this work demonstrates the utility of transparent, rule-based text mining approaches for large-scale bibliometric analyses in plant science. By leveraging standardized gene nomenclature and reproducible pipelines, similar analyses can be readily extended to other crop species or comparative genomic contexts. As genomic resources continue to expand, literature-scale mining approaches may play an increasingly important role in identifying research trends, uncovering understudied genomic regions, and informing strategic priorities in crop improvement research.

### Limitations and Future Work

This study has several limitations that should be considered when interpreting the results. First, the analysis was restricted to PubMed abstracts rather than full-text articles. Although abstracts typically summarize the principal findings of a study, additional gene mentions may appear in full-text sections, or methodological descriptions that were not captured in this analysis. Consequently, the reported frequencies likely represent conservative estimates of total gene reporting in the literature.

Second, the rule-based text mining approach relied on standardized *Glyma* gene identifiers. While soybean gene nomenclature is relatively consistent, some studies may reference genes using alternative symbols, aliases, or descriptive names without explicitly including the canonical *Glyma* identifier. Such cases would not be detected by the present method, potentially leading to underestimation of gene mentions.

Third, this study quantifies literature frequency rather than biological importance. Chromosome-level differences in gene mentions may reflect research trends, historical focus, funding priorities, or experimental accessibility rather than intrinsic genomic features such as gene density, recombination rates, or quantitative trait locus (QTL) distribution. The analysis does not incorporate normalization by chromosome length, annotated gene counts, or functional gene categories.

Fourth, contextual information surrounding gene mentions was not evaluated. The present approach identifies whether a gene is mentioned, but does not distinguish between experimental validation, review citation, or peripheral reference. More advanced natural language processing (NLP) methods, such as relation extraction or semantic role labeling, could provide deeper insight into how genes are discussed in the literature.

Future work could address these limitations by incorporating full-text mining, expanding entity recognition to include gene aliases and orthologs, and integrating genomic annotations from resources such as SoyBase [13]. Comparative analyses across crop species (e.g., maize or *Arabidopsis*) could reveal whether chromosome-level publication biases are conserved. Additionally, network-based analyses of co-mentioned genes may uncover clusters of functionally related genes and highlight understudied genomic regions.

## Figures and Tables

**Table 1:** Most frequently mentioned soybean genes in PubMed abstracts (2006–2025) and their functional descriptions. The table lists the top-ranked *Glyma* gene identifiers by the number of abstracts in which each gene was mentioned, together with brief gene descriptions obtained from SoyBase (www.soybase.org).

Gene_ID	Number of Abstracts	Description
Glyma.20G085100	4	CCT domain-containing protein; IPR010402 (CCT domain); GO:0005515 (protein binding)
Glyma.18G022500	4	Alpha-soluble NSF attachment protein 2; IPR000744 (NSF attachment protein); GO:0005515 (protein binding), GO:0006886 (intracellular protein transport)
Glyma.06G095100	2	MYB/SANT-like domain-containing protein; IPR024752 (Myb/SANT-like domain)
Glyma.19G193400	2	Basic leucine zipper (bZIP) transcription factor HBP-1a; IPR004827 (basic leucine zipper domain); GO:0003700 (DNA-binding transcription factor activity), GO:0043565 (sequence-specific DNA binding)
Glyma.19G194300	2	Flowering locus T (FT)-like protein; IPR008914 (phosphatidylethanolamine-binding protein, PEBP family)
Glyma.19G195400	2	Beta-fructofuranosidase (cell wall invertase); IPR001362 (glycoside hydrolase family 32), IPR008985 (lectin/glucanase superfamily), IPR023296 (beta-propeller domain); GO:0005975 (carbohydrate metabolic process)
Glyma.11G108300	2	Cytochrome P450 family protein; IPR001128 (cytochrome P450); GO:0005506 (iron ion binding), GO:0020037 (heme binding), GO:0055114 (oxidation–reduction process)
Glyma.09G284700	2	Peroxidase family protein; IPR010255 (heme peroxidase); GO:0004601 (peroxidase activity), GO:0006979 (response to oxidative stress), GO:0020037 (heme binding), GO:0055114 (oxidation–reduction process)
Glyma.03G227300	2	Phytochrome-like protein kinase; light-sensing photoreceptor protein; IPR001294 (phytochrome domain); GO:0000155 (sensor kinase activity), GO:0007165 (signal transduction), GO:0009584 (detection of visible light)
Glyma.16G149300	2	Cytochrome P450 family protein; IPR001128 (cytochrome P450); GO:0005506 (iron ion binding), GO:0020037 (heme binding), GO:0055114 (oxidation–reduction process)
Glyma.03G226000	2	Mannan endo-1,4-beta-mannosidase; IPR017853 (glycoside hydrolase superfamily); GO:0005975 (carbohydrate metabolic process)
Glyma.17G090200	2	RING-H2 zinc finger protein; IPR013083 (RING-type zinc finger); GO:0005515 (protein binding), GO:0008270 (zinc ion binding)
Glyma.05G243400	2	Elongation factor Tu (EF-Tu)-like GTP-binding protein; IPR000795 (GTP-binding domain), IPR027417 (P-loop NTP hydrolase); GO:0003924 (GTPase activity), GO:0005525 (GTP binding)
Glyma.14G194300	2	Fatty acid desaturase 8; IPR005804 (fatty acid desaturase); GO:0006629 (lipid metabolic process), GO:0055114 (oxidation–reduction process)
Glyma.09G171200	2	Pentatricopeptide repeat (PPR) protein; IPR002885 (PPR repeat); GO:0005515 (protein binding)
Glyma.04G167900	2	Light-harvesting chlorophyll a/b-binding protein; IPR022796 (chlorophyll-binding protein); GO:0016020 (membrane)
Glyma.07G102300	2	FAD/NAD(P)-binding oxidoreductase; IPR001327 (oxidoreductase NAD-binding domain); GO:0016491 (oxidoreductase activity), GO:0050660 (FAD binding), GO:0055114 (oxidation–reduction process)
Glyma.06G204300	2	TCP transcription factor family protein; IPR005333 (TCP domain)
Glyma.17G152300	2	Purine permease family protein; IPR004853 (transporter domain)
Glyma.02G304700	2	Phytochromobilin:ferredoxin oxidoreductase; IPR009249 (ferredoxin-dependent bilin reductase); GO:0010024 (phytochromobilin biosynthesis), GO:0055114 (oxidation–reduction process)

## Data Availability

The data analyzed in this study were derived from publicly available PubMed abstracts accessed via the National Center for Biotechnology Information (NCBI). The processed dataset, Python scripts, and Jupyter Notebook workflow used for gene extraction and analysis are publicly available in the corresponding author’s GitHub repository: https://github.com/abdelmajidk/NLP-Glyma-Soybean.

## Abbreviations

The following abbreviations are used in this manuscript:

**Abbreviation**	**Definition**
NLP	Natural Language Processing
QTL	Quantitative Trait Locus
GWAS	Genome-Wide Association Study
RNA-seq	RNA Sequencing
CRISPR	Clustered Regularly Interspaced Short Palindromic Repeats
NCBI	National Center for Biotechnology Information
PMID	PubMed Identifier
XML	Extensible Markup Language
GO	Gene Ontology
PPR	Pentatricopeptide Repeat
NSF	N-ethylmaleimide-Sensitive Factor
PEBP	Phosphatidylethanolamine-Binding Protein
CCT	CONSTANS, CONSTANS-like, and TOC1 domain
CSV	Comma-Separated Values
